# Effects of enzymes originating from *Trichoderma reesei* on the performance, organ index, serum biochemical indices, and intestinal health homeostasis of broiler chicken

**DOI:** 10.3389/fvets.2025.1550463

**Published:** 2025-05-21

**Authors:** Luyao Zhang, Yang Liu, Chunyuan Xie, Xuan Huang, Ping Deng, Chuang Li, Weican Wan, Qiuzhong Dai, Yan Hu, Yuyong Ma, Xu Zhang, Guitao Jiang

**Affiliations:** ^1^Department of Animal Nutrition, Hunan Institute of Animal Husbandry and Veterinary Medicine, Changsha, China; ^2^Department of Animal Nutrition, Yuelu Mountain Laboratory, Changsha, China; ^3^CJ International Trading CO., LTD, Shanghai, China; ^4^Department of Animal Nutrition, Hengyang Academy of Agricultural Sciences, Hengyang, China

**Keywords:** broiler chicken, enzyme system, growth performance, organ indices, serum biochemical parameters, gut microbiota

## Abstract

To study the effect of combined enzymes from *Trichoderma reesei* on growth performance, organ index, serum biochemical indices, and gut microbiota in broiler chickens, an experiment was conducted with 648 one-day-old AA broilers. The broilers were randomly divided into six groups. The control group was fed a corn-soybean meal basal diet, and the experimental groups were supplemented with different enzyme combinations. The experiment lasted 42 d. The results showed that from day 1–21, the average daily gain (ADG) of T1–T4 groups was higher than that of the control group (*P* = 0.005). From day 22–42, T2 had a higher ADG than T3 and T4 (*P* = 0.017). T1, T3, and T4 had a lower feed-to-gain ratio (F/G) than the control group from day 1–21 (*P* = 0.001).In terms of serum indices, T1–T5 had higher levels of triiodothyronine (T3), growth hormone (GH), and insulin-like growth factor-1 (IGF-1) at 21 and 42 days compared to the control group (*P* < 0.001), with some exceptions. T2–T5 had higher levels of superoxide dismutase (SOD), glutathione peroxidase (GSH-PX), and glutathione (GSH) at 21 and 42 days (*P* < 0.001), with some exceptions. For organ indices, T3 had a lower gizzard index and T5 had a higher gizzard index than the control group (*P* = 0.001), and T4 had a lower liver index than the control group (*P* = 0.041). Enzyme supplementation also changed the gut microbiota.In conclusion, all enzyme systems improved broiler growth performance from day 1–21. Only xylanase and the carbohydrase system from Trichoderma reesei enhanced growth from day 22–42. All enzyme systems enhanced antioxidant and immune capacities during the study period.

## 1 Introduction

Non-starch polysaccharidases (NSPases) have been widely used in poultry production in areas where grains being the main feed ingredient. However, little improvements in poultry production performance were observed with the addition of xylanase alone, or the combination of xylanase and β-glucanase, or xylanase, amylase, and protease (1–3). In this regard, Wu et al. (4) noted that enzyme addition improved the nutrient digestibility of wheat diets and fermentability of arabinoxylans, but maize diets were less affected, which might be related to the complexity of maize arabinoxylans as well as the high nutrient value of maize. Analysis of the NSP composition between maize and soybean meal showed that the NSP in wheat was mainly arabinoxylan with high solubility and few side chains, but the NPS in maize had a more complex composition, consisting mainly of arabinoxylan glucuronate with low solubility and a high degree of branched chains. Therefore, it is often necessary to increase the enzyme variety or dosage to achieve the improved performance in broiler. However, there are not many types of commercial monoenzymes available, and most of them are endonucleases. For this reason, the development directions of enzyme system for corn-soybean meal-based feed were fell into the following two paths. One was the introduction of new monoenzymes such as side-chain enzyme for xylan (arabinofuranosidase), which were found effectively improve the response level of enzymes system to the corn-soybean meal-based feed (2, 4). But the cost of their application was high and the available reaction substrates were few. The other path was the introduction of enzyme systems such as the natural enzyme systems from some filamentous fungi (*Trichoderma reesei* and *Aspergillus niger*, etc.), which were found significantly improve the production performance of animals fed corn-soybean meal based diets (5). The advantage of microbial enzyme systems over monoenzymes could be that they were developed to be more complete in composition and function during the evaluation of microbiota (6, 7).

Recent studies have further delved into the application of NSPases in poultry, especially broiler production. A study by Kouzounis et al. (8) discovered that a specific blend of NSPases, tailored to the unique NSP structure in corn-soybean meal diets, could enhance the energy utilization efficiency of broilers. By optimizing the proportion of xylanase, amylase, and protease, they found that the metabolizable energy of the feed increased by approximately 5–8%, leading to a significant improvement in the growth rate of broilers. This was mainly attributed to the more efficient breakdown of complex NSPs, making more nutrients accessible for absorption (8).

Moreover, research on the enzyme system from Trichoderma reesei has also advanced. New findings suggest that not only does this enzyme system improve the growth performance of broilers, but it also has a positive impact on their immune function. A 2025 study demonstrated that broilers fed with diets containing Trichoderma reesei enzyme system had a 15 - 20% higher antibody titer against common poultry pathogens compared to the control group. This indicates that the enzyme system might be modulating the gut–immune axis, enhancing the overall health of the broilers.

In terms of the industry, these research findings hold great practical significance. First, improving the growth performance of broilers directly increases the economic returns of poultry farmers. With enhanced nutrient digestibility and growth rate, more meat can be produced with the same amount of feed, reducing the cost per unit of production. Second, the positive effect on immune function means fewer incidences of diseases, reducing the need for antibiotics and other medications. This not only benefits the health of the poultry but also aligns with the growing consumer demand for antibiotic–free poultry products, enhancing the competitiveness of poultry products in the market. Third, the development of more effective enzyme systems allows for the better utilization of corn-soybean meal-based feeds, which are widely available and cost-effective feed ingredients. This helps to optimize the feed formulation, making the poultry industry more sustainable and resource-efficient.

Therefore, the present study was conducted to investigate the effects of combinations of xylanase, amylase, protease, and enzyme system originated from Trichoderma reesei in corn-soybean meal type diets on the growth performance, organ index, serum biochemical indexes, and intestinal microbiota composition of broilers, with a view to providing further data for the application of Trichoderma reesei enzyme system on broiler chickens.

## 2 Materials and methods

### 2.1 Materials and reagents

The enzymes used in the experiment were supplied by CJ Youtell (Shandong) Biotechnology Co., Ltd.

### 2.2 Animals and experiment design

All experimental procedures implemented in this study were meticulously reviewed and approved in accordance with the guidelines of the Animal Welfare and Ethics Committee of the Hunan Institute of Animal Husbandry and Veterinary Medicine (Approval No.: HIAVS - IACUC - 2023 - 05). A total of 648 healthy broilers, with an initial body weight of 46.16 ± 0.49 g, were selected for this experiment, which adopted a completely randomized group design. The broilers were allocated into six treatment groups, each consisting of nine replicates with 12 broilers per replicate. The control group (CON) was fed a corn-soybean meal-based basal diet. The enzyme-treated groups (T1, T2, and T3) were provided with the basal diet supplemented with different enzyme combinations. Specifically, the diet for Group T1 was the basal diet supplemented with 20,000 U/g xylanase (derived from Trichoderma reesei). The diet for Group T2 was the basal diet supplemented with 20,000 U/g xylanase and the Trichoderma reesei enzyme system (with the main components being 6,500 U/g cellulase and 45,000 U/g glucanase). The diet for Group T3 was the basal diet supplemented with 20,000 U/g xylanase, the Trichoderma reesei enzyme system, 550 U/g medium-temperature amylase, and 150,000 U/g alkaline protease. The wheat-bran-treated groups (T4 and T5) were fed the experimental diet of Group T2, in which 2% and 4% of the corn was replaced with wheat bran, respectively. The experiment had a duration of 42 days.

The broilers were housed in closed tree-layer cages. The facility was cleaned, disinfected and fumigated before the experiment. During the experiment, the broilers were provided with 24 h of light and ad-liberum feed and water. The disinfection and immunization were conducted according to routine procedures. The broilers were fed powdery diets from 1 to 21-d-old, and pelleted diets from 22 to 42-d-old. The composition and nutrient levels for the basal diet were shown in Table 1.

**Table 1:** Composition and nutrient levels of experimental diets (%, as-fed basis).

**Composition and nutrient levels of experimental diets (%, as-fed basis)Ingredients, %**	**1–21 d**	**22–42 d**
Corn	48.53	49.49
Flour	8	8
Puffed Soya	7	7
Soybean meal (43%)	31	26.2
Soybean oil	1.000	5
Salt	0.300	0.3
Limestone	1.520	1.34
Dicalcium phosphate	1.360	1.14
Lysine sulfate	0.530	0.5
DL-Methionine 98%	0.230	0.21
L-Tryptophan 99%	0.010	0
L-Threonine 98%	0.100	0.13
Bentonite	0.000	0.3
Mildew inhibitor	0.150	0.15
Antioxidant	0.010	0.01
Choline chloride 50%	0.100	0.1
Trace mineral premix^a^	0.030	0.03
Multivitamin^b^	0.100	0.1
High temperature resistant phytase	0.020	0.02
Probiotics (100 billion/g)	0.010	0.01
Total	100.00	100.00
Nutrient name		
Metabolizable Energy Mcal/kg^c^	2.88	3.15
Ca	1.05	0.91
CP	22.03	20.04
p	0.68	0.68
Non-phytate phosphorus	0.37	0.32
Crude fiber	4.95	4.68
Lysine	1.40	1.26
DL-Methionine	0.53	0.49
L-Threonine	0.89	0.84
L-Tryptophan	0.25	0.22

^a^Trace mineral premix provided the following per kg of diets: Cu (as copper sulfate)10 mg, Fe (as ferrous sulfate)80 mg, Mn (as manganese sulfate)90 mg, Zn (as zinc sulfate)93 mg, I(as potassium iodide)0.40 mg.

^b^Multivitamin for poultry provided the following per kg of diets: VA 5 000 IU, VB1 12 mg,VB2 15 mg, VB5 40 mg, VB6 4 mg, VB12 0.02 mg, VD3 800 IU, VE 20 IU, VK30.5 mg, biotin 0.2 mg, folic acid 0.6 mg, D-pantothenic acid 60 mg, nicotinic acid 60 mg.

^c^Except for CP and CF, which are measured values, other nutrients are calculated values.

### 2.3 Sample collection

#### 2.3.1 Collection and test of feed samples

Two hundred grams feed ingredient and diets samples were collected according to GB/T14699.1-2005 “feed sampling method”, placed into labeled bags, and kept at −20°C for testing.

#### 2.3.2 Growth performance

The body weight (Body weight, BW) of broilers was measured at each replicate on days 1, 21, and 42 of the feeding trial, and the feed intake of broilers was recorded on a replicate basis. The average daily gain (Average daily gain, ADG), average daily feed intake (Average daily feed intake, ADFI), and feed-to-gain ratio (Feed to gain ratio, F/G) were calculated for the periods of 1–21 days, 22–42 days, and the entire trial period using the following formulas:

ADG = (Final weight – Initial weight)/Number of trial days

ADFI = (Amount of feed provided – Amount of feed remaining)/Number of trial days

F/G = ADFI/ADG

#### 2.3.3 Serum samples

On the 21 d and 42 d, one broiler was taken from each replicate after 12 h fasten, and 10 mL blood was collected from the wing vein with a vacuum blood collection tube. After 1 h being kept in room temperature, the blood samples were centrifuged at 3,000 r/min for 15 min to retrieve the serum samples, which were moved into 1.5 mL sterilized EP tubes, and stored at −20°C for testing.

#### 2.3.4 Organ samples

On the 42 d, the sampling broilers were slaughtered after blood collection and the gizzard, glandular stomach, pancreas, liver and heart were weighted. And the organ index was calculated as the organ weight divided by the body weight.

#### 2.3.5 Feces samples

After organ sampling, the cecal chyme of each sampling broilers were collected into 2 mL EP tubes and kept in −80°C for testing.

### 2.4 Assay of serum biochemical, antioxidant, and immunological parameters

The detection of the biochemical parameters in serum was carried out using an automatic biochemical analyzer (Shanghai Kehua Bio-engineering Co., Ltd., Shanghai, China) with the spectrophotometric method (dual wavelength and post-spectrophotometric), which included the concentrations of total protein (TP), triglyceride (TG), urea nitrogen (UREA), glucose (GLU), and creatine kinase (CK).

The thiobarbituric acid method was used to determine the concentrations of malondialdehyde (MDA), superoxide dismutase (SOD), glutathione peroxidase (GSH-PX), and glutathione (GSH) in serum, which were the antioxidant parameters. Enzyme-linked immunosorbent assay (ELISA) was used to determine the concentrations of immunoglobulin G (IgG), immunoglobulin A (IgA), immunoglobulin M (IgM) in serum, which were the immunological parameters. The kits were provided by Nanjing Jiancheng Bioengineering Company (Nanjing, China). A GC-2010 Immuno-Counter (Anhui USTC Zonkia Scientific Instruments, Hefei, China) with radioimmunoassay was employed to determine the concentrations of triiodothyronine (T3), tetraiodothyronine (T4), growth hormone (GH), and insulin-like growth factor-1 (IGF-1) in serum. The reagent kits were provided by Tianjin Jiuding Medical Biological Engineering Company, Tianjin, China.

### 2.5 High-throughput sequencing analysis of 16S rRNA in feces microbiome

Total DNA extraction of feces microbial genome: 0.5 g of feces samples were weighed, and the total DNA of the microbiome was extracted by a QIAamp DNA kit; the extraction process was performed referring to the instruction manual of the kit, the step of magnetic bead striking was added, and the extracted DNA was run through agarose gel electrophoresis for quality detection.

PCR amplification of the V3-V4 region of the 16S rRNA sequence and library construction: This step was performed by Shanghai Meiji Biomedical Technology Co. (Shanghai China). The V3-V4 region of the 16S rRNA gene was amplified by PCR using specific primers with barcodes. To ensure the reliability and accuracy of the amplified sequences, the number of amplification cycles for each sample was ensured to be the same. The PCR-amplified products were then purified, quantified, and homogenized, and sequencing libraries were created. After the libraries passed the quality control check, microbial diversity sequencing analysis was carried out using an Illumina HiSeq 2500.

After splicing and filtering the readings of each sample, OTUs (operational taxonomic units) were clustered using UPARSE (version 7.11, http://drive5.com/uparse/, accessed on 3 February, 2024), and chimeric sequences were identified and removed using UCHUIME software, (http://drive5.com/usearch/manual/uchime_algo.html, accessed on 9 February 2024). The phylotype classification analysis of the Silva (SSU123) 16S rRNA database was performed using the RDPv2.2 classification program with a confidence threshold of 70%. Further α-diversity analysis and β-diversity analysis were performed; the 16S rRNA gene-sequencing results were applied to the I-sanger platform to predict species annotation, composition, variation, and the functional prediction of bacterial communities in piglet feces. Wayne plots were used to evaluate the distribution of the OTUs among different groups. Unweighted UniFrac distances were measured to determine differences in microbial communities. Finally, selected environmental factors were correlated with microorganisms for heat map analysis.

### 2.6 Data analysis

The experimental data were preliminarily calculated, processed, and organized using Excel, and analyzed using the One-Way ANOVA program in SPSS 22.0. Duncan's multiple range test was employed for significance testing. The results were expressed as “mean ± SD”. *P* < 0.05 indicated significant differences, while *P* < 0.01 indicated extremely significant differences.

16S rRNA analysis in microbiomics: QIIME 2.0 software was employed to analyze species α-diversity indices, including the Shannon and Chao-1 indices, to assess species richness and diversity. Based on the phylogenetic relationships among Operational Taxonomic Units (OTUs), principal coordinates analysis (PCoA) using UniFrac distance matrices was conducted to calculate β-diversity between samples, evaluating similarities among different communities. Through Linear discriminant analysis (LDA), the Linear discriminant analysis effect size (LEfSe) was utilized for inter-group significance differential analysis, highlighting taxonomic groups with LDA scores ≥ 2 where a higher LDA value indicates greater differences between groups. Spearman correlation analysis was applied to examine the interrelationships between microbial samples and various environmental factors.

## 3 Results

### 3.1 Growth performance

As shown in Table 2, the body weights (BWs) of broilers in T1, T2, T3, and T4 at 21-d-old of age were significantly higher than those in the control group (CON) (*P* < 0.05). Additionally, at 42 days of age, the BW of broilers in group T2 was significantly higher than that in groups CON, T1, T3, and T4 (*P* < 0.05). From day 1 to day 21, the average daily weight gains (ADGs) of broilers in groups T1, T2, T3, and T4 were significantly higher than those in the CON group (*P* < 0.05). Moreover, the F/G of broilers in groups T1, T3, and T4 were significantly improved compared to the CON group (*P* < 0.05). From day 22 to day 42, the ADG of broilers in T2 was significantly higher than that in groups T3 and T4 (*P* < 0.05). The F/G of broilers in groups T2, T3, T4, and T5 were lower than those in groups CON and T1, although these differences were not statistically significant (*P* > 0.05). Over the entire experimental period (day 1 to day 42), the ADG of broilers in group T2 was significantly higher than that in all other groups except T5 (*P* < 0.05). The F/G of broilers in the CON group were higher than those in the other groups, but these differences were not statistically significant (*P* > 0.05).Throughout all stages, there were no significant differences in the average daily feed intake (ADFI) among all groups (*P* > 0.05).

**Table 2:** Effects of different enzyme combinations on broiler performance.

**Item**	**CON**	**Treatment**					***P* value**

		**T1**	**T2**	**T3**	**T4**	**T5**	
**BW (g)**							
1 d	46.14 ± 0.51	46.11 ± 0.45	46.17 ± 0.47	46.16 ± 0.44	46.14 ± 0.47	46.06 ± 0.70	0.998
21 d	567.23 ± 20.50^c^	600.68 ± 30.97^ab^	597.76 ± 38.55^ab^	601.39 ± 25.30^ab^	627.66 ± 29.54^a^	582.00 ± 36.76^bc^	0.004
42 d	2,241.77 ± 92.43^b^	2,266.76 ± 52.67^b^	2,361.70 ± 51.48^a^	2,216.04 ± 79.59^b^	2,215.48 ± 78.77^b^	2,279.11 ± 158.11^ab^	0.031
**ADG (g/d)**							
1–21 d	27.01 ± 0.98^c^	28.60 ± 1.48^ab^	28.19 ± 1.91^ab^	28.64 ± 1.21^ab^	29.89 ± 1.41^a^	27.71 ± 1.75^bc^	0.005
22–42 d	79.99 ± 4.2^ab^	79.66 ± 2.53^ab^	83.78 ± 5.26^a^	76.89 ± 3.48^b^	76.57 ± 3.06^b^	80.62 ± 6.83^ab^	0.017
1–42 d	52.4 ± 3.27^b^	52.87 ± 1.25^b^	54.89 ± 2.71^a^	51.66 ± 1.9^b^	51.65 ± 1.87^b^	53.17 ± 3.77^ab^	0.022
**ADFI (g/d)**							
1–21 d	39.23 ± 1.84	38.98 ± 1.17	39.45 ± 1.94	38.29 ± 1.99	38.70 ± 1.61	39.51 ± 3.19	0.796
22–42 d	115.39 ± 10.42	109.97 ± 7.98	116.14 ± 14.52	106.63 ± 4.95	106.98 ± 8.13	106.86 ± 14.10	0.193
1–42 d	77.31 ± 5.56	74.48 ± 3.70	77.79 ± 7.08	72.46 ± 2.80	72.84 ± 3.77	73.18 ± 6.44	0.120
**F/G (g/g)**							
1–21 d	1.45 ± 0.07^c^	1.36 ± 0.06^ab^	1.40 ± 0.08^bc^	1.34 ± 0.06^ab^	1.30 ± 0.08^a^	1.43 ± 0.12^bc^	0.001
22–42 d	1.45 ± 0.14	1.45 ± 0.22	1.39 ± 0.10	1.39 ± 0.10	1.42 ± 0.14	1.35 ± 0.29	0.816
1–42 d	1.48 ± 0.12	1.45 ± 0.14	1.42 ± 0.12	1.40 ± 0.08	1.41 ± 0.09	1.39 ± 0.19	0.700

Results are expressed as mean ± standard error. ^a–*d*^Peer data with different shoulder labels (lowercase English letters) indicate significant differences (P < 0.05).

### 3.2 Serum biochemical parameters

As shown in Table 3, the different enzyme combinations did not significantly affect the serum concentrations of TP, TG, UREA, GLU and CK in broilers at either 21 or 42 days of age (*P* > 0.05). At both 21 and 42 days of age, the serum concentrations of T3, GH, and IGF-1 in broilers of T3 were significantly higher than those in theCON group (*P* < 0.05). There were no significant differences in the serum concentrations of T4 among groups at either 21 or 42 days of age (*P* > 0.05).

**Table 3:** Effect of different enzyme combinations on serum biochemical indices of 21 d and 42 d broilers.

**Item**	**CON**	**Treatment**					***P* value**

		**T1**	**T2**	**T3**	**T4**	**T5**	
**21 d**							
TP (g/L)	104.71 ± 8.81	111.90 ± 9.74	108.06 ± 10.67	103.44 ± 11.17	102.43 ± 5.72	107.13 ± 9.73	0.315
TG (mmol/L)	1.35 ± 0.54	1.67 ± 0.57	1.70 ± 0.61	1.74 ± 0.56	1.49 ± 0.81	0.96 ± 0.35	0.059
UREA (mmol/L)	6.11 ± 1.84	5.53 ± 1.35	6.62 ± 1.50	5.82 ± 1.50	5.26 ± 1.44	7.19 ± 2.06	0.147
GLU (mmol/L)	8.43 ± 2.29	8.35 ± 2.72	8.22 ± 1.55	8.00 ± 2.47	9.82 ± 1.77	8.00 ± 2.41	0.525
CK (U/L)	171.89 ± 103.73	157.67 ± 113.07	228.33 ± 91.01	235.56 ± 90.46	195.67 ± 89.15	188.44 ± 89.10	0.482
T3 (nmol/L)	3.82 ± 0.69^c^	5.18 ± 0.84^b^	5.29 ± 0.65^ab^	5.63 ± 0.88^ab^	5.92 ± 0.65^a^	5.97 ± 0.61^a^	< 0.001
T4 (nmol/L)	118.42 ± 13.80	144.84 ± 28.00	105.83 ± 19.67	133.48 ± 27.82	123.38 ± 34.41	119.49 ± 32.85	0.067
GH (ng/mL)	7.97 ± 1.05^c^	9.73 ± 1.67^b^	9.93 ± 1.43^b^	10.97 ± 1.49^b^	10.93 ± 1.64^b^	12.82 ± 1.29^a^	< 0.001
IGF-I (ng/mL)	85.88 ± 15.75^c^	107.98 ± 17.44^b^	110.66 ± 15.75^b^	107.72 ± 16.69^b^	116.01 ± 13.97^ab^	128.12 ± 19.00^a^	< 0.001
**42 d**							
TP (g/L)	105.48 ± 8.54	105.18 ± 12.61	103.96 ± 11.27	105.66 ± 12.28	102.72 ± 7.95	105.23 ± 11.18	0.992
TG (mmol/L)	1.03 ± 0.51	1.59 ± 0.73	1.32 ± 0.59	1.72 ± 0.56	1.44 ± 0.57	1.66 ± 0.52	0.141
UREA (mmol/L)	6.14 ± 1.77	6.89 ± 1.58	5.57 ± 1.86	5.23 ± 1.61	7.15 ± 1.74	6.30 ± 2.23	0.206
GLU (mmol/L)	8.54 ± 1.84	8.02 ± 1.76	8.57 ± 1.89	8.64 ± 2.31	9.36 ± 2.22	9.49 ± 2.42	0.672
CK (U/L)	148.56 ± 82.20	176.22 ± 79.69	176.67 ± 102.91	161.89 ± 103.56	214.22 ± 82.97	177.67 ± 98.47	0.766
T3 (nmol/L)	4.24 ± 0.50^c^	5.52 ± 0.60^b^	5.68 ± 0.65^b^	6.14 ± 0.67^ab^	6.03 ± 0.58^b^	6.67 ± 0.75^a^	< 0.001
T4 (nmol/L)	130.82 ± 31.80	127.22 ± 30.36	120.98 ± 30.01	116.42 ± 38.14	101.61 ± 22.09	119.94 ± 28.77	0.427
GH (ng/mL)	9.34 ± 1.38^b^	11.62 ± 0.87^a^	9.46 ± 1.50^b^	11.55 ± 1.32^a^	11.56 ± 1.83^a^	12.78 ± 1.64^a^	< 0.001
IGF-I (ng/mL)	97.47 ± 12.68^c^	117.04 ± 14.80^b^	114.57 ± 17.65^b^	135.49 ± 14.16^a^	127.00 ± 16.47^ab^	130.71 ± 19.17^a^	< 0.001

^a–*c*^Peer data with different shoulder labels (lowercase English letters) indicate significant differences (*P* < 0.05).

### 3.3 Serum antioxidant parameters

As shown in Table 4, for 21-day-old broilers, the serum concentrations of SOD, GSH-PX and GSH were significantly higher in groups T2, T3, T4, and T5 compared to the CON group (*P* < 0.05). Additionally, the serum concentration of MDA was significantly lower in groups T3, T4, and T5 compared to the CON group (*P* < 0.05). Among the treatment groups, the serum concentrations of MDA in the bran-treated groups (T4 and T5) were significantly lower than those in groups T1 and T2 (*P* < 0.05). For 42-day-old broilers, the serum concentrations of SOD, GSH-PX, and GSH in groups T2, T3, T4, and T5 were significantly higher than those in the CON group (*P* < 0.05). In contrast, the serum MDA concentration was significantly higher in the CON group compared to the other groups (*P* < 0.05). Additionally, the serum concentrations of GSH-PX and GSH in the wheat bran-treated groups (T4 and T5) were significantly higher than those in the enzyme-treated groups (T1, T2, and T3) (*P* < 0.05).

**Table 4:** Effect of different enzyme combinations on serum antioxidant indexes of 21 d and 42 d broilers.

**Item**	**CON**	**Treatment**					***P* value**
		**T1**	**T2**	**T3**	**T4**	**T5**	
**21 d**							
MDA (nmol/mL)	8.22 ± 0.90^a^	8.21 ± 1.02^a^	7.58 ± 1.27^ab^	7.01 ± 1.14^bc^	6.40 ± 1.02^cd^	5.63 ± 0.87^d^	< 0.001
SOD (ng/mL)	180.94 ± 43.80^c^	220.54 ± 21.99^bc^	239.81 ± 42.78^a^	251.08 ± 32.64^a^	240.94 ± 35.18^a^	267.45 ± 39.81^a^	< 0.001
GSH-Px (ng/mL)	112.59 ± 16.88^c^	152.48 ± 19.54^ab^	146.63 ± 12.79^b^	153.45 ± 27.40^ab^	154.80 ± 21.63^ab^	171.19 ± 18.20^a^	< 0.001
GSH (mmol/L)	166.99 ± 44.38^c^	222.27 ± 42.09^b^	233.37 ± 31.71^ab^	251.33 ± 47.44^ab^	236.36 ± 37.22^ab^	268.11 ± 55.26^a^	< 0.001
**42 d**							
MDA (nmol/mL)	9.48 ± 1.26^a^	7.26 ± 1.22^bc^	8.02 ± 1.16^b^	7.02 ± 1.20^bc^	6.82 ± 1.24^c^	6.90 ± 1.15^bc^	< 0.001
SOD (ng/mL)	185.98 ± 27.19^b^	275.46 ± 41.26^a^	271.45 ± 32.97^a^	272.69 ± 32.18^a^	285.75 ± 25.87^a^	297.46 ± 37.49^a^	< 0.001
GSH-Px (ng/mL)	125.57 ± 13.47^c^	143.40 ± 21.52^bc^	143.08 ± 19.18^bc^	161.29 ± 21.27^ab^	162.02 ± 21.82^a^	174.67 ± 17.05^a^	< 0.001
GSH (mmol/L)	201.91 ± 35.47^d^	237.41 ± 42.78^c^	247.12 ± 34.14^bc^	262.36 ± 44.92^bc^	279.53 ± 39.19^ab^	345.95 ± 16.60^a^	< 0.001

^a–*d*^Peer data with different shoulder labels (lowercase English letters) indicate significant differences (*P* < 0.05).

### 3.4 Serum immunological parameters

As shown in Table 5, the serum concentrations of IgG, IgA, and IgM in 21-day-old broilers, and of IgA and IgM in 42-day-old broilers, were significantly higher in the CON group compared to the other treatment groups (*P* < 0.05). In contrast, the serum concentration of IgG in 42-day-old broilers was significantly higher in groups T1, T3, and T5 compared to the CON group (*P* < 0.05).

**Table 5:** Effect of different enzyme combinations on serum immunological indices of 21 d and 42 d broilers.

**Item (μg/mL)**	**CON**	**Treatment**					***P* value**

		**T1**	**T2**	**T3**	**T4**	**T5**	
**21 d**							
IgG	1,289.83 ± 215.14^c^	1,564.64 ± 251.56^b^	1,524.45 ± 239.47^b^	1,591.06 ± 231.74^b^	1,505.61 ± 201.80^bc^	1,893.80 ± 227.60^a^	< 0.001
IgA	170.57 ± 26.64^c^	209.83 ± 29.91^b^	212.81 ± 33.86^b^	223.95 ± 29.54^b^	233.50 ± 31.1^2ab^	257.02 ± 31.42^a^	< 0.001
IgM	261.41 ± 61.08^d^	404.11 ± 73.99^bc^	337.22 ± 80.61^c^	429.34 ± 81.80^b^	421.69 ± 63.72^b^	515.85 ± 70.85^a^	< 0.001
**42 d**							
IgG	1,438.20 ± 164.72^d^	1,684.79 ± 218.05^bc^	1,591.17 ± 323.70^cd^	1,947.43 ± 197.83^a^	1,633.75 ± 201.78^bcd^	1,842.92 ± 192.70^ab^	< 0.001
IgA	182.11 ± 35.87^c^	232.43 ± 29.06^b^	213.19 ± 28.95^b^	239.89 ± 28.35^b^	228.03 ± 30.84^b^	280.47 ± 18.55^a^	< 0.001
IgM	375.85 ± 67.94^c^	456.17 ± 86.18^b^	473.89 ± 83.38^b^	462.13 ± 80.93^b^	475.11 ± 85.53^ab^	550.29 ± 73.43^a^	0.002

^a–*d*^Peer data with different shoulder labels (lowercase English letters) indicate significant differences (*P* < 0.05).

### 3.5 Organ index

As shown in Table 6, there were no significant differences in the organ indexes of the glandular, spleen, pancreas, and heart among the groups of 42-day-old broilers (*P* > 0.05). Compared to the CON group, the gizzard index was significantly lower in group T3 and significantly higher in group T5 (*P* < 0.05). Additionally, the liver index of broilers in group T4 was significantly lower than that in the other groups (*P* < 0.05).

**Table 6:** Effect of different enzyme combinations on organ index of 21 d and 42 d broilers.

**Item**	**CON**	**Treatment**					***P* value**
		**T1**	**T2**	**T3**	**T4**	**T5**	
Gizzard	9.57 ± 1.25^b^	9.44 ± 1.02^bc^	9.24 ± 0.78^bc^	8.48 ± 1.26^c^	8.57 ± 0.79^bc^	10.94 ± 1.29^a^	0.001
Glandular	4.21 ± 0.86	4.04 ± 0.83	4.32 ± 0.69	3.92 ± 0.52	3.55 ± 0.86	4.38 ± 0.80	0.264
Spleen	1.08 ± 0.80	0.91 ± 0.26	0.97 ± 0.38	0.96 ± 0.42	1.02 ± 0.41	0.90 ± 0.29	0.966
Pancreas	1.82 ± 0.32	1.66 ± 0.56	1.73 ± 0.32	1.80 ± 0.28	1.76 ± 0.30	1.68 ± 0.27	0.904
Liver	26.46 ± 3.53^a^	26.98 ± 2.64^a^	27.07 ± 2.44^a^	26.02 ± 3.03^a^	23.22 ± 1.35^b^	27.80 ± 2.84^a^	0.041
Heart	3.80 ± 0.52	3.62 ± 0.55	3.57 ± 0.33	4.12 ± 0.61	4.15 ± 0.42	3.63 ± 0.38	0.054

^a–*c*^Peer data with different shoulder labels (lowercase English letters) indicate significant differences (*P* < 0.05).

### 3.6 Microbiological composition of gut microbiota

#### 3.6.1 Diversity of intestinal microflora

**Figure 1:**
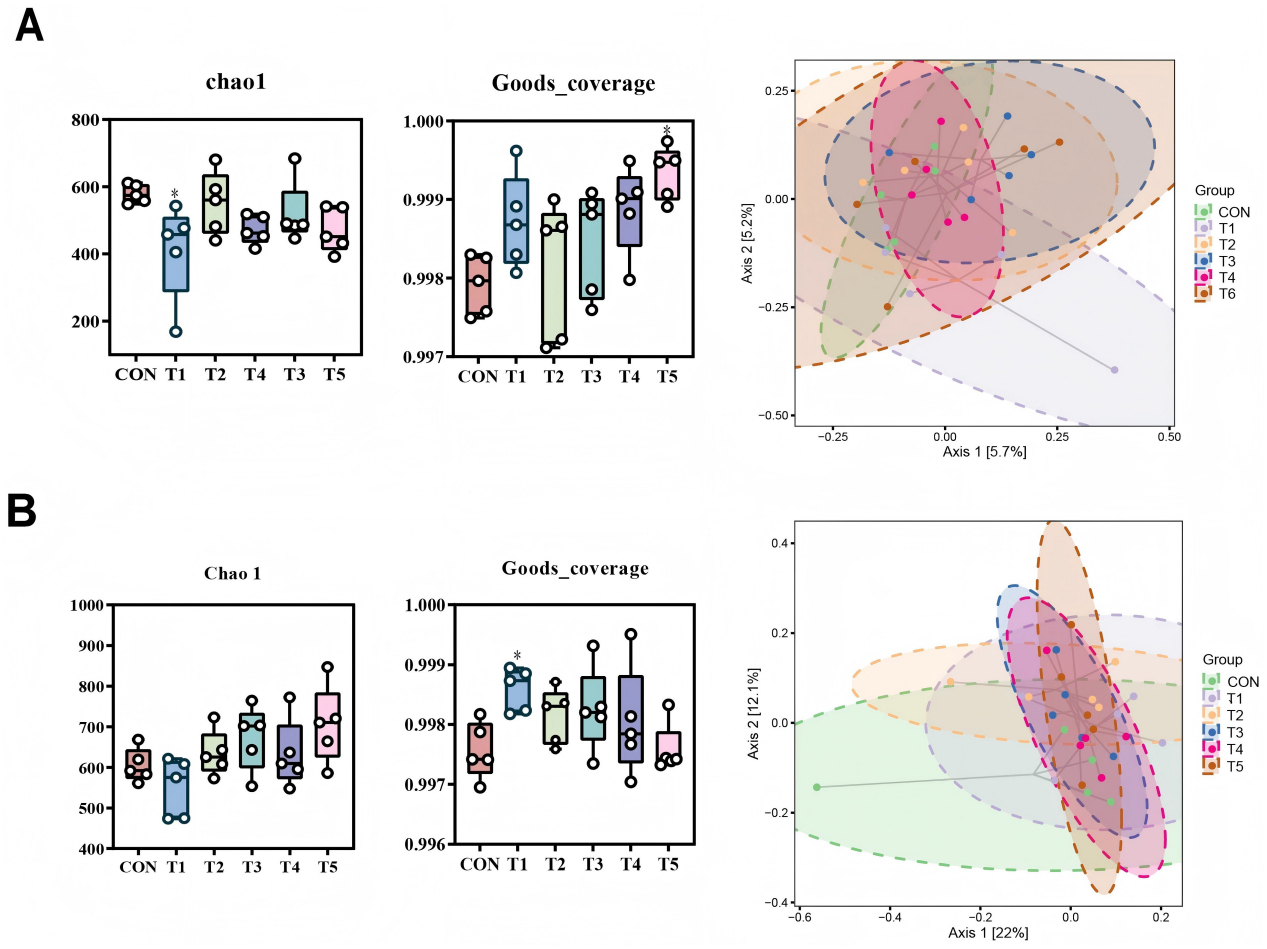
Effects of different enzyme combinations on the diversity of intestinal microflora in 21-d-old **(A)** and 42-d-old **(B)** broilers.*means differ.

The richness and diversity of the microbial communities were evaluated by α-diversity indices (Chao-1 and Goods_coverage). At 21-d-old, the Chao-1 index in T1 was reduced and the Goods_coverage index in T5 was increased comparing to CON (*P* < 0.05, Figure 1A). At 42-d-old, the Goods_coverage index in T1 was increased comparing to CON (*P* < 0.05, Figure 1B). PCoA analysis at OUT level showed that the intestinal microbial compositions of broilers at 21-d-old or 42-d-old were not clearly separated (Figures 1A, B).

#### 3.6.2 Gut microbiota composition

**Figure 2:**
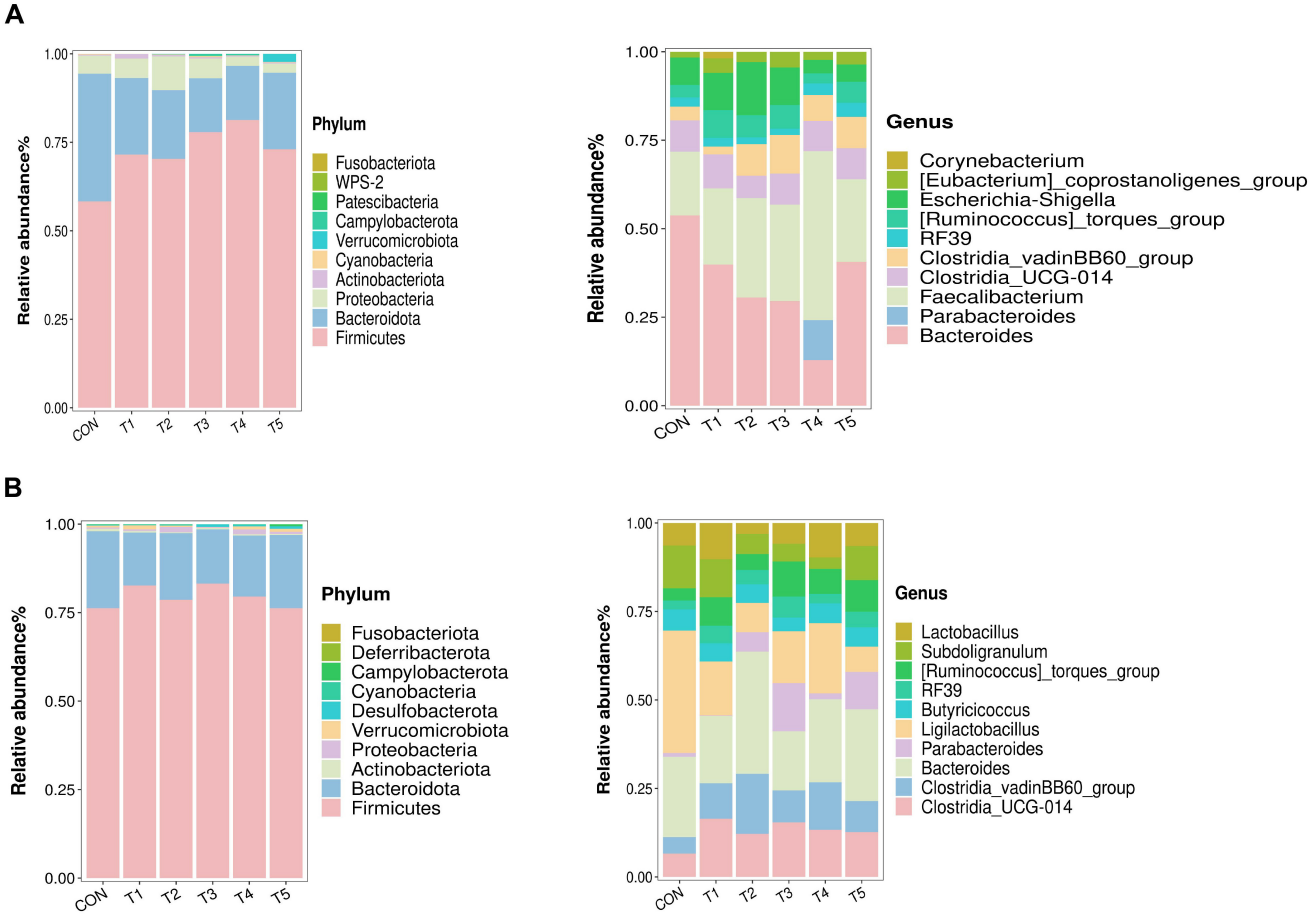
Microbiological composition of intestinal contents in 21-d-old **(A)** and 42-d-old **(B)** broilers.

As shown in Figure 2A, the intestinal microbiota in 21-d-old broilers were mainly Firmicutes, Bacteroidota, and Proteobacteria at the phylum level, which contributed to more than 90% of the whole microflora. And the top 10 dominant genus were *Bacteroides, Parabacteroides, Faecalibacterium, Clostridia_UCG-014, Clostridia_vadinBB60_group, RF39, [Ruminococcus]_toraues_group, Escherichia-Shigella, [Eubacterium]_coprostanoligenes_group, Corynebacterium*.

As shown in Figure 2B, the dominant microbial phyla in 42-d-old broilers were Firmicutes and Bacteroidota, and the top 10 dominant genera were *Clostridia_UCG-014, Clostridia_vadinBB60_group, Bacteroides, Parabacteroides, Ligilactobacillus, Butyricoccus, RF39, [Ruminococcus]_toraues_group, Subdoligranulum*, and *Lactobacillus*.

#### 3.6.3 LEfSe analysis of gut microbiota in broilers

**Figure 3:**
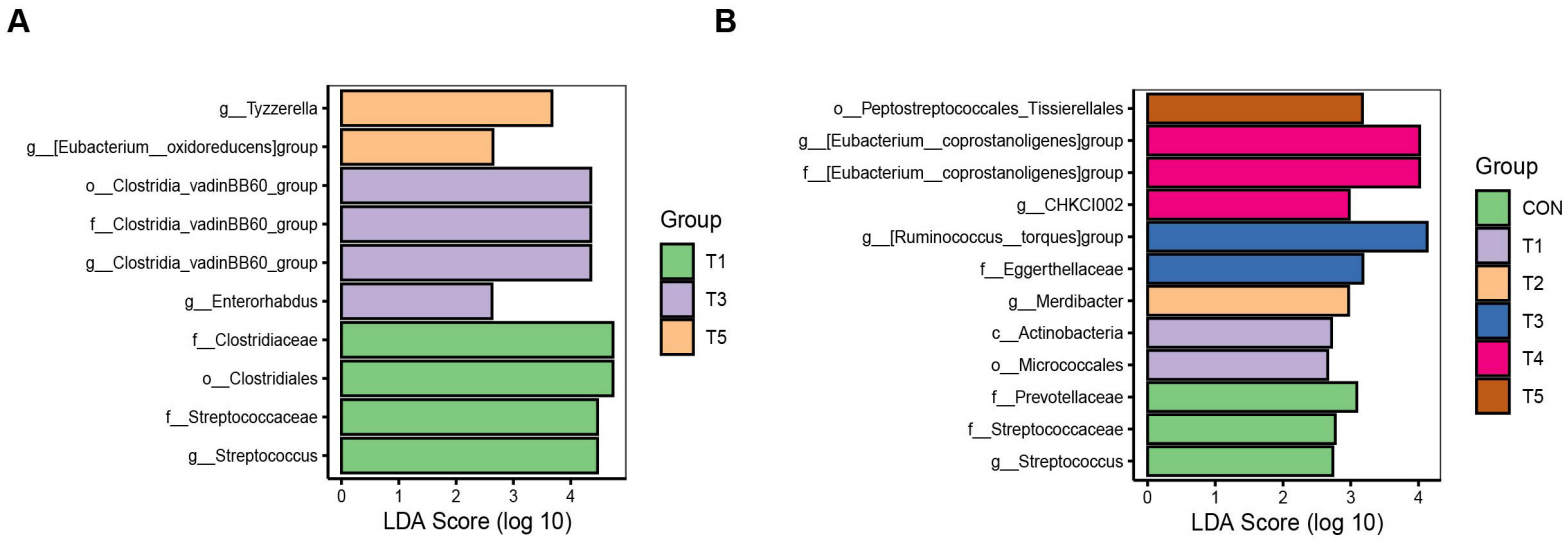
Microbiological LEfSe analysis of intestinal contents flora in 21-d-old **(A)** and 42-d-old **(B)** broilers. ^*^P < 0.05 and ^**^P < 0.01.

LEfSe analysis was applied to identify the differential taxa whose average relative abundances were higher than 0.1%. For 21-d-old broilers, *g_Streptococcus* (belonged to *f_Streptococcaceae*) and *o_Clostridiales* (belonged to *f_Clostridiaceae*) were enriched in T1; *g_Enterorhabdus* and *g_Clostridia_vadinBB60_group* (belonged to *f_Clostridia_vadinBB60_group, o_Clostridia_vadinBB60_group*) were enriched in T3; and *g_[Eubacterium_oxidoreducens] group* and *g_Tyzzerella* were enriched in T5 (LDA >2, *P* < 0.05, Figure 3A). For 42-d-old broilers, *g_Streptococcus* (belonged to *f_Streptococcaceae*) and *f_Prevotellaceae* were enriched in CON; *o_Micrococcales* and *c_Actinobacteria* were enriched in T1; *g_Merdibacter* was enriched in T2; *f_Eggerthellaceae* and *g_[Ruminococcus_torques]group* were enriched in T3; *g_CHKCI002* and *g_[Eubacterium_coprostanoligenes] group* (belonged to *f_[Eubacterium_coprostanoligenes] group*) were enriched in T4; and *o_Peptostreptococcales_Tissierellales* was enriched in T5 (LDA >2, *P* < 0.05, Figure 3B).

#### 3.6.4 Correlation analysis between gut microbiota and serum parameters in broilers

**Figure 4:**
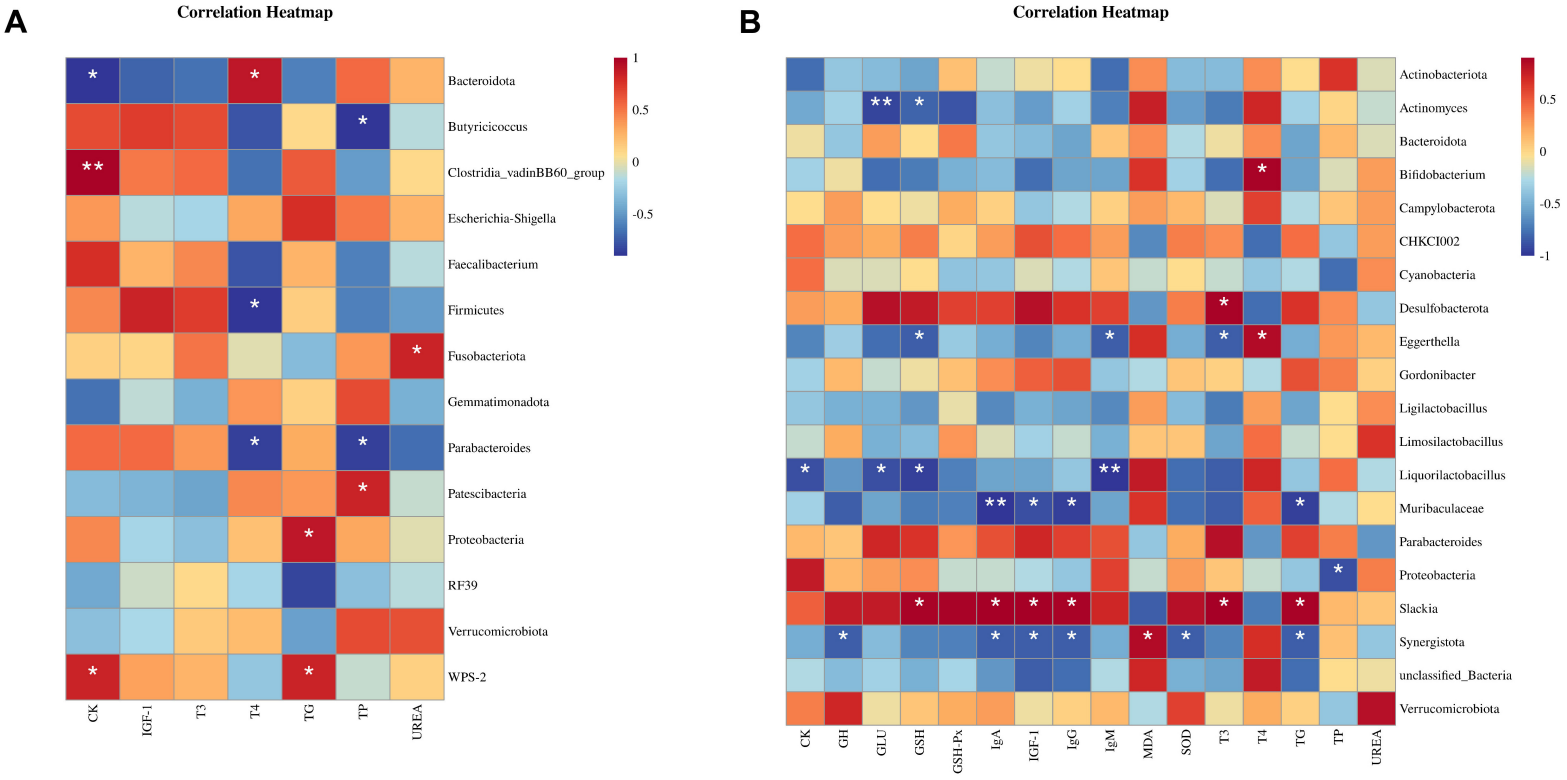
Correlation analysis between intestinal contents and environmental factors in 21-d-old **(A)** and 42-d-old **(B)** broilers.

As can be seen in Figure 4A, for the gut microbiota in 21-d-old broilers, the abundance of *Bacteroidota* showed a positive correlation with T4 level and a negative correlation with CK level in serum (*P* < 0.05); the abundance of *Butyricoccus* showed a negative correlation with TP level in serum (*P* < 0.05); the abundance of *Clostridia_vadinBB60_group* had a positive correlation with CK level in serum (*P* < 0.05); the abundance of Firmicutes had a negative correlation with T4 level in serum (*P* < 0.05); the abundance of *Fusobacteriota* had a positive correlation with UREA level in serum (*P* < 0.05); the abundance of *Parabacteroides* was negatively correlated to T4 and TP levels in serum (*P* < 0.05); the abundance of *Patescibacteria* was positively correlated to serum level of TP (*P* < 0.05); the abundance of *Proteobacteria* was positively correlated to serum level of TG (*P* < 0.05); and the abundance of *WPS-2* was positively correlated to the serum levels of CK and TG (*P* < 0.05).

As can be seen in Figure 4B, for the gut microbiota in 42-d-old broilers, the abundance of Actinomyces showed negative correlations with serum levels of GLU and GSH (*P* < 0.05); the abundance of Bifidobacterium showed a positive correlation with serum level of T4 (*P* < 0.05); the abundance of Desulfobacterota showed a positive with serum level of T3 (*P* < 0.05); the abundance of Eggerthella showed a positive correlation with serum levels of T4 and negative correlations with serum levels of GSH, IgM, and T3 (*P* < 0.05); abundance of Liquorilactobacillus showed negative correlations with serum levels of CK, GLU, GSH, and IgM (*P* < 0.05); the abundance of Muribaculaceae was negatively correlated with serum levels of IgA, IGF-1, IgG and TG (*P* < 0.05); the abundance of Proteobacteria was negatively correlated with serum level of TP (*P* < 0.05); the abundance of Slackia was positively correlated with serum levels of GSH, IgA, IGF-1, IgG, T3, and TG (*P* < 0.05); and the abundance of Synergistota was positively correlated with serum level of MDA and negatively correlated with serum levels of GH, IgA, IGF-1, IgG, SOD, and TG (*P* < 0.05).

## 4 Discussion

### 4.1 Effect of different enzyme combinations on growth performance of broiler

The application of NSP in cereal-based diets has been widely recognized. However, when xylanase only, or a combination of xylanase and β-glucanase, or a combination of xylanase, amylase, and protease were applied to corn-soybean meal-based diets, the improvements in poultry production performance were minimal (1–3). Kouzounis et al. (8) studied the effects of adding a single xylanase to wheat-soybean meal or corn-soybean meal diets on the intestinal oligosaccharide composition and other metabolites. It was found that the enzyme addition improved the digestibility of nutrients and the fermentability of arabinoxylan in wheat diets but had little influence on corn diets. This result could be caused by the complexity of corn arabinoxylan and the high nutritional value of corn. Recently, a new enzymes system was found, which was produced by modified Trichoderma reesei by knocking out the protease. The main components of the enzyme system were cellulases (including cellobiohydrolase, β-1,4-glucanase, β-glucosidase), β-1,3-1,4-glucanase, and xyloglucanase. But the xylanase activity in the enzyme system was low, and protease and amylase activities were not detected (9). Xylan is the most important hemicellulose component of cereal grains, accounting for approximately 50% of its NSP (10). Therefore, the direct use of the new enzyme system in poultry diets could have certain flaws. This study compared the effects of diets supplemented with several enzyme combinations on broilers, aiming to determine the optimistic strategy for the utilization of the new enzyme system.

Recently, a novel enzyme system has been identified, which is produced by a genetically modified strain of *Trichoderma reesei* with protease genes knocked out. The primary components of this enzyme system include cellulases (comprising cellobiohydrolase, β-1,4-glucanase, and β-glucosidase), β-1,3-1,4-glucanase, and xyloglucanase. However, the xylanase activity in this enzyme system is relatively low, and no detectable activities of protease and amylase were observed. Xylan is the most important hemicellulose component in cereal grains, accounting for approximately 50% of the NSP content. Therefore, the direct application of this novel enzyme system in poultry diets may have certain limitations.

In this study, all broilers in all treatment groups except T5 had improved BW and ADG comparing to CON during 1-21 d period. And only broilers in T2 had improved BW and ADG during the entire feeding period. In addition, the growth performances of broilers in two bran substitution groups (T4 and T5) were not different from CON. With sufficient nutrient levels, most studies showed that adding enzyme combination to corn-soybean meal diets reduced feed intake in broilers but improved nutrient digestibility and feed conversion ratio (11–14). However, Wang et al. (15) found that supplementing xylanase to corn-soybean meal-based or wheat-soybean meal-based diets significantly improved feed intake in broilers from 1 to 42 d. Despite minimal changes in feed intake, T2 significantly improved the BW at 42 d and ADG over the entire feeding period, confirming the effectiveness of the Trichoderma reesei enzyme system in breaking down the NSP in corn-soybean meal diets. It was reported that the applications of other microbial enzyme systems in corn-based diets, such as the mixed enzyme system from Aspergillus niger and Trichoderma reesei (16) and the enzyme system from Penicillium funiculosum (17), also effectively enhanced poultry performance. Furthermore, it was found in this study that although the feed intake of broilers decreased when 2% and 4% wheat bran was substituted for corn and supplemented with xylanase + Trichoderma reesei enzyme system, the final BW at 42 d and F/G of broilers were still improved. This result might be attributed to the fact that wheat bran was easily hydrolyzed into xylo-oligosaccharides, which compensated the deficiency of xylo-oligosaccharides as in corn-soybean meal diets, and mitigated the negative effects of reduced dietary energy levels (2, 8).

### 4.2 Effect of different enzyme combinations on serum biochemical and antioxidant indices of broiler

UREA is a metabolic product of protein metabolism in animal bodies, while TG and TP are crucial components of blood lipids. The concentration of GLU in serum reflects the intestinal absorption status and is directly correlated with the digestibility of dietary carbohydrates. Creatine kinase, mainly existing in skeletal muscles, cardiac muscles, and brain tissues, is a significant kinase directly related to intracellular energy transport, muscle contraction, and ATP regeneration. In this experiment, different enzyme combinations had no significant effect on the concentrations of TP, TG, UREA, GLU, and CK in the serum of broilers throughout the experimental period. This result was inconsistent with a previous study, that after adding xylanase to the corn-wheat-soybean meal diet of broilers, the serum TP content and ALB activity increased, while the levels of TG and AST in serum decreased significantly (18). This might be related to the addition of more wheat in Chen's study, which increased the intestinal viscosity of broilers and improved the absorption and metabolism of nutrients. T3 and T4 are hormones widely involved in regulating body metabolism, modulating the metabolism of serum glucose, fats, and proteins. GH redistributes absorbed nutrients among tissues and promotes the growth of bones, cartilage, and tissues. IGF-1, a polypeptide hormone synthesized by the liver, promotes growth and development, cell proliferation and differentiation, and plays a vital role in the immune system. This study found that all enzyme combinations significantly increased the concentrations of T3, GH, and IGF-1 in the serum of broilers. This result was similar to that of Derakhshan et al. (18) and Kim et al. (19), which confirmed that the supplement of enzyme combination improved the growth performance of broilers.

Under normal physiological conditions, the body maintains a dynamic equilibrium between free radicals and its antioxidant system. However, under stress, microbial invasion, illness, aging, or specific physiological situations, an increase in free radicals occurs. The enzymatic antioxidant system comprises SOD and CAT. SOD eliminates superoxide radicals, mitigating their damage to cell membranes. Meanwhile, CAT converts hydrogen peroxide (H_2_O_2_) into H_2_O. MDA is the primary degradation product of lipid peroxidation. In this study, compared to the CON, the concentrations of SOD, GSH-PX, and GSH in the serum of broilers from all treatment groups except T1 were elevated, while the concentrations of MDA were reduced. Notably, the serum GSH concentrations of broilers in the later stage (22–42 d) with 4% bran substitution (T5) were higher than that of the unsubstituted group (CON, T1, T2, and T3), while the serum MDA concentration was lower. Similarly, previous studies found that adding NSPase or proteases to piglet diets could enhance antioxidant enzyme activity and reduce serum MDA concentrations (20, 21). These results suggest that NSPase may reduce the generation of free radicals in the body or enhance antioxidant levels. In this regard, Zeng et al. (22) evaluated the antioxidant effects of different polysaccharides from cereals and grains, and discovered that more than 10 types of polysaccharides possessed scavenging abilities against DPPH (1,1-diphenyl-2-picrylhydrazyl), ABTS [2,2′-azinobis(3-ethylbenzothiazoline-6-sulfonic acid)], and hydroxyl radicals, inferring that this scavenging ability might be related to polysaccharide-phenolic compounds. Additionally, xylanase broke down xylan in the diet into xylooligosaccharides, which could modulate the structure of intestinal microbial communities, enhance the volatile fatty acid production, and improve the antioxidant status of weaned broilers (23, 24). The optimal serum antioxidant levels observed in the 4% bran substitution group (T5) in this study might be attributed to the easier degradation of bran into oligomers compared to corn.

### 4.3 Effect of different enzyme combinations on serum immunological parameters of broiler

Immunoglobulins (Ig) are glycoproteins produced by B cells in response to antigenic stimulation. They serve as the primary effector molecules of the immune system, responsible for recognizing and binding specific antigens to exert immune defense functions. Their main function is to bind antigens to form antigen-antibody complexes, thereby blocking the invasion of pathogens into the body. Immunoglobulins also play a crucial role in mucosal immunity, especially IgA, which is the predominant immunoglobulin on mucosal surfaces and can neutralize pathogens to maintain mucosal homeostasis (25).

Long et al. (21) found that after feeding broilers with diets containing different enzyme combinations for 42 d, the concentrations of IgA and IgG in broiler serum were significantly increased. Similarly, enzyme combination also elevated the levels of serum IgA, IgG, and IgM in piglets, which might be related to the ability of xylanase and β-glucanase to decompose NSP in corn-soybean meal-barley diets into oligosaccharides (26). Consistent with these findings, the current study also observed that throughout the experimental period, the serum concentrations of IgA and IgM in broilers from all experimental groups were higher than those in the control group. This suggests that enzyme combinations may enhance the immune function and health status of broilers by modulating the gut microbiota and promoting the production of immunoglobulins.

### 4.4 Effect of different enzyme combinations on organ index of broiler

The normal development of animal internal organs is fundamental to the proper functioning of various physiological processes within the animal body, with organ indices serving as critical indicators of organ health and function. In the current study, the incorporation of different enzyme combinations into the diet did not elicit significant differences in the organ indices of the glandular stomach, spleen, pancreas, or heart of broilers. Notably, compared to the control group (CON), the gizzard index of broilers in treatment group T3 was reduced, while that in group T5 was increased. Additionally, the liver index of broilers in group T4 was decreased.These findings are consistent with those of Wickramasuriya et al. (27), who reported that supplementing broiler diets with exogenous emulsifiers and multi-enzyme combinations for 21 days did not significantly influence the organ weights of broilers. This suggests that the tested enzyme combinations and additives may not have a substantial impact on the organ development of broilers under the experimental conditions employed. Future research may explore the potential long-term effects of these additives on organ health and function, as well as their interactions with other dietary components.

### 4.5 Effect of different enzyme combinations on microbiota composition of broilers

The gut microbiota is a critical determinant of animal health and disease defense, functioning as a virtual metabolic organ that influences digestion, metabolism, immune regulation, and neurological function. Dysbiosis, or imbalances in the gut microbiota, has been implicated in various diseases, including inflammatory bowel disease, obesity, and colorectal cancer. In the current study, the addition of different enzyme combinations to the diets of broilers significantly influenced the α-diversity indices of the gut microbiota. Specifically, compared to the control group (CON), the Chao-1 index of gut microbiota in 21-day-old broiler chickens was decreased, indicating reduced species richness, while the Goods_coverage index was increased, suggesting improved sampling coverage. By 42 days, the Goods_coverage index of gut microbiota was further increased, highlighting the dynamic changes in microbial diversity over time. These results demonstrate that enzyme supplementation can modulate the diversity of the gut microbiota in broilers. To elucidate the effects of different enzyme combinations on the gut microbiota structure, bacterial taxa with an average relative abundance >0.01% across all samples were selected for analysis. Consistent with previous findings, the gut microbiota of broilers at both 21 and 42 days was primarily composed of the phyla *Firmicutes* and *Bacteroidota*. These phyla are known to play a dominant role in the gut microbiota of poultry, influencing nutrient absorption and overall health. The observed changes in microbial diversity and composition suggest that enzyme supplementation may enhance the functional capacity of the gut microbiota, potentially improving digestive efficiency and immune function in broilers. Future research should explore the specific mechanisms by which these enzyme combinations influence microbial ecology and host health outcomes (28).

The phylum Firmicutes has been extensively documented for its pivotal role in energy metabolism and gut health maintenance. Numerous studies have reported that the abundance of Firmicutes increases in the gut of broilers fed diets supplemented with enzyme combinations, which aligns with the findings of the current study. This suggests that enzyme supplementation can enhance gut health by promoting the growth of beneficial Firmicutes species (29, 30).

Bacteroidetes, another dominant phylum in the gut microbiota, is recognized for its capacity to inhibit the colonization of intestinal pathogens, thereby contributing to host health. Scaldaferri et al. (31) demonstrated that inflammation in the human intestine is associated with a decrease in Bacteroidetes abundance. However, in the present study, no significant differences were observed in the abundance of Bacteroidetes between the control group (CON) and the treatment groups in broiler chickens. This indicates that the enzyme combinations used in this study did not significantly impact the abundance of Bacteroidetes.

At the genus level, the gut microbiota of broiler chickens at both 21 and 42 days was primarily composed of *Bacteroides, Parabacteroides, Faecalibacterium, Clostridia_UCG-014, and Clostridia_vadinBB60_group*. *Bacteroides* is known for its ability to enhance animal growth performance by improving carbohydrate metabolism and utilization. *Faecalibacterium*, particularly *Faecalibacterium prausnitzii*, has been identified as a key player in reducing and mitigating intestinal inflammation, contributing to gut homeostasis. *Parabacteroides*, on the other hand, is recognized for its role in regulating the host's mucosal immune system, reducing inflammation, and participating in carbon metabolism. Collectively, these findings highlight the multifaceted roles of gut microbiota in modulating host health and metabolism. Future research should focus on elucidating the specific mechanisms through which these microbial taxa interact with the host and influence health outcomes (32–34).

In this study, the relative abundance of Bacteroides in broilers fed diets supplemented with enzyme combinations was significantly lower, while the abundances of Parabacteroides and Faecalibacterium were higher compared to the control group (CON). These findings are consistent with previous research (35–38). The present study has identified several key bacterial taxa associated with immunity and antioxidant capacities in broilers, including *Bacteroidota, Butyricicoccus, Clostridia_vadinBB60_group, Firmicutes*, and *Parabacteroides*.

Furthermore, the abundance of *Slackia* was positively correlated with serum levels of GSH, IgA, IGF-1, IgG, T3, and TG, while the abundance of Muribaculaceae was negatively correlated with serum levels of IgA, IGF-1, IgG, and TG. *Slackia* is an important component of the intestinal microecology, contributing to the maintenance of gut microbial balance and exerting a positive impact on broiler health. In contrast, Muribaculaceae are conditionally pathogenic bacteria that are highly associated with the development of inflammatory bowel disease. This aligns with recent findings that the proportion of Slackia is positively related to IgA and IgG levels in broilers (39, 40).

These results indicate that feeding enzyme combinations can synergistically improve gut microbial balance, diversity, and abundance in broiler chickens, thereby promoting gut health. This highlights the potential of enzyme supplementation as a strategy to enhance both the microbiota-mediated immune function and overall health of broilers (41–43). Future research should focus on elucidating the specific mechanisms through which these microbial taxa interact with the host and influence health outcomes.

## 5 Conclusion

In summary, when fed with corn-soybean meal diets, the addition of different enzyme combinations in the early stage promoted the growth of broilers, improved their antioxidant and immunological capabilities, and modulated the gut microbiota composition. However, in the later stage and throughout the entire feeding period, only the combination of xylanase and Trichoderma reesei enzyme system showed a positive response. Nevertheless, all enzyme treatment groups improved the antioxidant capacity and immune ability of broiler chickens.

## Data Availability

The data presented in the study are deposited in the NCBI, accession number PRJNA1202383.
